# Cost Variance in Patients With Soft Tissue Sarcoma Who Develop Postoperative Wound Complications

**DOI:** 10.5435/JAAOSGlobal-D-21-00147

**Published:** 2021-07-07

**Authors:** Benjamin K. Wilke, Jeannie Buckner, Maria T. Huayllani, Aaron C. Spaulding, Peter M. Murray, Antonio J. Forte

**Affiliations:** From the Department of Orthopedic Surgery, Mayo Clinic, Jacksonville, FL (Dr. Wilke, Dr. Buckner, Dr. Murray, and Dr. Forte); the Division of Plastic Surgery, Mayo Clinic (Dr. Huayllani and Dr. Forte); and Robert D. and Patricia E. Kern Center for the Science of Health Care Delivery, Mayo Clinic (Dr. Huayllani, Dr. Spaulding, and Dr. Forte).

## Abstract

**Background:**

Wound complications after a soft-tissue sarcoma surgery are common, occurring in up to 30% to 40% of patients who undergo preoperative radiation therapy. Although risk factors for developing complications are well-known, there is a paucity of literature on the increased healthcare costs after a wound complication. The purpose of this study was to detail these additional costs after a soft-tissue sarcoma surgery.

**Methods:**

A retrospective review of 99 patients from January 2013 to October 2019 was performed. Hospital and professional charges for the primary surgical procedure and any subsequent hospitalization or procedure related to a wound complication were compiled. Costs were inflated to 2019 dollars.

**Results:**

Total costs were 21.3% higher for patients who developed a wound complication (*P* = 0.006). Most patients (32 of 42; 76.2%) who developed a complication required a return trip to the operating room. The average number of return trips was 1.2 (range 0 to 5). For each return trip to the operating room because of a wound complication, an associated increased overall cost of 13.2% was noted (*P* < 0.001).

**Conclusion:**

Wound complications after a soft-tissue sarcoma resection are common and add considerable expense to the episode of care. A reduction in wound complications may markedly decrease the cost of treating soft-tissue sarcomas and continues to be an opportunity for improvement.

Wound complications after a soft-tissue sarcoma resection are common, reported to occur in up to 30% to 40% of patients. Predominant causes of postoperative wound complications are due to wound dehiscence and infection.^1^ These complications often require repeat trips to the operating room to treat an infection or perform flaps for wound coverage.

Attempts have been made to mitigate postoperative complications without notable progress.^1,2,3,4,5,6^ Many studies have reported on risk factors for developing complications, most of which are nonmodifiable.^7,8^ In contrast, a paucity of literature exists, evaluating the additional healthcare costs that result from dealing with these postoperative wound complications in sarcoma surgery.

This study's primary objective was to compare the overall healthcare costs for patients who underwent resection of a soft-tissue sarcoma and developed a postoperative wound complication relative to patients who did not develop a complication. We hypothesized that there would be a markedly higher cost for patients who developed a postoperative wound complication.

## Methods

After institutional review board approval, a retrospective review of patients who underwent a soft-tissue sarcoma resection at our institution from January 2013 to October 2019 was performed. Patient demographics, tumor characteristics, and wound complications were collected from chart review. Wound complications were defined as postoperative infection and/or wound dehiscence.

The number of clinic visits to the orthopaedic surgery and plastic surgery teams involved in the procedure was calculated over the first postoperative year. Repeat hospitalizations and unplanned operations because of a wound complication were recorded.

Costs analyzed included the hospital and professional charges for the initial surgical procedure and any subsequent hospitalization or operation because of a wound complication. These costs were provided for each patient by our institution's financial office. In each case, costs were inflated to 2019 dollars using the gross domestic product: implicit price deflator.^9^ In addition, cost to charge ratios were collected from the cost reports, and inflated costs were multiplied by the cost to charge ratio to create the dependent variable of interest.

Outpatient visits were not calculated in the cost analysis because of accounting complexity for the 90-day global period. The average number of outpatient visits was simply compared between patients who did and did not develop a wound complication to evaluate the time commitment required for both patient and providers while treating a wound complication.

Descriptive statistics were used to summarize patients and tumor characteristics; continuous variables were summarized using mean and standard deviation, whereas categorical variables were summarized using frequency and percentages. A Student *t*-test was used for comparison of means of numeric variables. A Fisher test and chi-square tests were used to compare the percentage of categorical variables. Multiple linear regression was used to identify associations between patients and tumor characteristics and the total operation costs, including hospital and professional charges during each admission. The following were included as patient-level covariates: patient age, sex, body mass index, smoking status, antecedent use of steroids, the presence of diabetes, peripheral vascular disease, coronary artery disease, preoperative albumin, radiation therapy, the location of the tumor, depth of tumor, tumor size, the type of wound closure, and wound complication status or the number of returns to the operating room because of wound complication.

Two separate models were run. The first sought to identify the associations of the number of return trips to the operating room for wound complications with cost. The second was to identify associations with the presence of a wound complication and cost. Owing to data skewness, total cost was log-transformed to meet normal distribution requirements for regression. Parameter estimates and 95% confidence intervals were subsequently exponentiated to identify percent change in cost for ease of interpretation.

## Results

In total, 99 patients were included in the study. The average age was 60 years (24 to 88 years). Fifty-five men (55.6%) and 44 women (44.4%) were involved. Seventy-six patients (76.8%) received preoperative radiation. Patient demographics are listed in Table 1.

**Table 1:** Descriptive Analysis of Patients Who Underwent Sarcoma Resection

	No Wound Complication (N = 57)	Wound Complication (N = 42)	Total (N = 99)	*P* Value^a^
Mean age (SD)	57.8 (17.0)	63.1 (16.8)	60.0 (17.0)	0.12
Sex, n (%)				0.54
Women	27 (47.4)	17 (40.5)	44 (44.4)	
Men	30 (52.6)	25 (59.5)	55 (55.6)	
Mean BMI (SD)	28.1 (6.9)	29.9 (6.9)	28.9 (6.9)	0.19
Smoking, n (%)				0.44
No smoking	33 (57.9)	21 (50)	54 (54.5)	
Former or active smoking	24 (42.1)	21 (50)	45 (45.5)	
Steroid use, n (%)				0.69
No	52 (91.2)	40 (95.2)	92 (92.9)	
Yes	5 (8.8)	2 (4.8)	7 (7.1)	
Diabetes mellitus, n (%)				0.07
No	53 (93)	33 (78.6)	86 (86.9)	
Yes	4 (7)	9 (21.4)	13 (13.1)	
PVD, n (%)				0.39
No	55 (96.5)	38 (90.5)	93 (93.9)	
Yes	2 (3.5)	4 (9.5)	6 (6.1)	
CAD, n (%)				0.23
No	47 (82.5)	30 (71.4)	77 (77.8)	
Yes	10 (17.5)	12 (28.6)	22 (22.2)	
Preoperative albumin, n (%)				**0.04**
<4.0	4 (7)	10 (23.8)	14 (14.1)	
≥4	36 (63.2)	24 (57.2)	60 (60.6)	
Unknown	17 (29.8)	8 (19)	25 (25.3)	
Radiation therapy, n (%)				0.09
No	17 (29.8)	6 (14.3)	23 (23.2)	
Yes	40 (70.2)	36 (85.7)	76 (76.8)	
Location, n (%)				**<0.001**
Axial	5 (8.8)	3 (7.1)	8 (8.1)	
Lower extremity	30 (52.6)	37 (88.1)	67 (67.7)	
Upper extremity	22 (38.6)	2 (4.8)	24 (24.2)	
Depth of tumor, n (%)				0.77
Deep to fascia	50 (87.7)	36 (85.7)	86 (86.9)	
Superficial to fascia	7 (12.3)	6 (14.3)	13 (13.1)	
Tumor size, n (%)				0.78
≤5 cm	20 (35.1)	12 (28.6)	32 (32.3)	
>5 cm	36 (36.2)	29 (69)	65 (65.7)	
Unknown	1 (1.8)	1 (2.4)	2 (2)	
Closure, n (%)				**0.03**
Primary closure	30 (52.6)	12 (28.6)	42 (42.4)	
Free flap	6 (10.5)	7 (16.7)	13 (13.1)	
Local flap	20 (35.1)	18 (42.9)	38 (38.4)	
No flap-STSG	1 (1.8)	5 (11.9)	6 (6.1)	
Mean of no. of returns to OR because of wound complication (SD)	0 (0)	1.2 (1.1)	0.5 (0.9)	**<0.001**

BMI = body mass index, CAD = coronary artery disease, OR = operating room, PVD = peripheral vascular disease, STSG = split-thickness skin graft

a Fisher or chi-square and Student *t*-test for categorical and numerical variables, respectively.

Bold = P < 0.05.

When comparing patients with and without a postoperative wound complication, a higher percentage of patients with tumors located in the lower extremity who developed a postoperative wound complication (*P* < 0.001). Similarly, more patients who developed wound complications required flap coverage during their index procedure (*P* = 0.03) and had a preoperative albumin <4.0 g/dL (*P* = 0.04). In addition, although not reaching statistical significance, a trend toward more patients in the wound complication cohort with diabetes was observed (*P* = 0.07). A similar nonsignificant trend with a higher rate of preoperative radiation therapy in the wound complication cohort was noted (*P* = 0.09).

Most patients who developed a postoperative wound complication required an additional operation (32 of 42, 76.2%). The average number of additional operations was 1.2 (range 0 to 5). The average duration of wound care required until the wound had healed was 5.7 months (SD 5.3) and required an average of 8 postoperative outpatient visits (SD 4) during the first year, compared with an average of 5 outpatient visits (SD 3) in the cohort that did not develop a wound complication.

Total costs were 21.3% higher for patients who developed a wound complication (*P* = 0.006; Table 2). When comparing individual variables, costs were 9.5% higher for former or active smokers than patients who did not smoke (*P* = 0.04). In addition, after a wound complication, total costs were 59.7% higher for patients who had undergone a free flap closure and 29.1% higher for patients who had undergone a local flap closure than patients who underwent primary closure during the index surgery (*P* < 0.001 and *P* = 0.001, respectively).

**Table 2:** Multiple Linear Regression for Costs by Wound Complication

	Estimate (Percent Change)	95% CI Lower	95% CI Upper	*P* Value
Age at surgery (yrs)	−0.2	−0.5	0.2	0.407
Men(vs women)	−1.1	−13.2	12.6	0.862
BMI	−0.5	−1.5	0.5	0.362
Former or active smoker (vs no smoker)	**9.5**	**0.4**	19.4	**0.04**
Steroid (vs no steroid use)	12.1	−10.0	39.5	0.3
Diabetes (vs no diabetes)	12.5	−10.0	40.6	0.294
PVD (vs no PVD)	−7.0	−29.5	22.9	0.607
CAD (vs no CAD)	1.1	−14.8	19.8	0.901
Albumin ≥4 (vs <4)	−6.7	−22.0	11.6	0.443
Radiation (vs no radiation)	6.2	−9.6	24.7	0.457
Location (axial vs lower extremity)	−11.7	−27.7	7.8	0.216
Location (upper vs lower extremity)	−0.2	−15.5	17.8	0.976
Deep (vs superficial)	−13.9	−29.9	5.7	0.148
Tumor size >5 cm (vs tumors ≤5 cm)	6.9	−6.2	21.9	0.311
Free flap closure at index procedure	**59.7**	**30.5**	**95.6**	**<0.001**
Local flap closure at index procedure	**29.1**	**11.2**	**49.8**	**0.001**
STSG at index procedure	9.2	−17.5	44.5	0.532
Wound complication (vs no wound complication)	**21.3**	**5.9**	**39.1**	**0.006**

BMI = body mass index, CAD = coronary artery disease, CI = confidence interval, PVD = peripheral vascular disease, STSG = split-thickness skin graft

Flap closure and STSG is compared with primary closure.

Bold = P < 0.05.

When costs were analyzed based on the number of additional operations due to wound complications, we observed a 48.6% increased cost for patients who had a free flap closure compared with those who had a primary closure during the index procedure (*P* < 0.001; Table 3). Similarly, local flaps had an increased cost of 29.3% compared with primary closure during the index procedure (*P* < 0.001). For each additional operation due to a wound complication, an associated increased cost of 13.2% was noted (*P* < 0.001).

**Table 3:** Multiple Linear Regression for Costs by the Number of Returns to OR because of Wound Complication

	Estimate (Percent Change)	95% CI Lower	95% CI Upper	*P* Value
Age at surgery (yrs)	−0.1	−0.4	0.3	0.694
Men (vs women)	1.4	−10.4	14.8	0.819
BMI	−0.2	−1.1	0.8	0.713
Former or active smoker (vs no smoker)	8.3	−0.2	17.5	0.055
Steroid (vs no steroid use)	18.3	−3.7	45.4	0.108
Diabetes (vs no diabetes)	12.3	−8.9	38.5	0.27
PVD (vs no PVD)	−0.2	−23.4	30.0	0.989
CAD (vs no CAD)	0.2	−14.7	17.6	0.984
Albumin ≥4 (vs <4)	−5.5	−20.2	11.9	0.506
Radiation (vs no radiation)	9.4	−5.6	26.9	0.228
Location (axial vs lower extremity)	−8.7	−24.5	10.3	0.339
Location (upper vs lower extremity)	−4.2	−17.3	11.0	0.562
Deep (vs superficial)	−9.2	−25.0	10.0	0.315
Tumor size >5 cm (vs tumors ≤5 cm)	8.0	−4.6	22.1	0.22
Free flap closure at index procedure	**48.6**	**21.9**	**81.1**	**<0.001**
Local flap closure at index procedure	**29.3**	**12.8**	**48.4**	**<0.001**
STSG at index procedure	4.0	−20.4	35.7	0.771
No. of return trips to OR due to wound complications	**13.2**	**6.3**	**20.6**	**<0.001**

BMI = body mass index, CAD = coronary artery disease, CI = confidence interval, OR = operating room, PVD = peripheral vascular disease, STSG = split-thickness skin graft

Flap closure and STSG is compared with primary closure.

Bold = P < 0.05.

## Discussion

An estimated 13,100 patients will be diagnosed with a soft-tissue sarcoma in the United States in 2020.^10^ Standard of care includes radiation and surgical resection. Depending on institutional preferences, many of these patients will undergo preoperative radiation therapy before tumor resection. It has been shown that 30% to 40% of patients who receive preoperative radiation will develop a postoperative wound complication.^1,11,12^

Owing to the high rate of wound complications in this patient population, many previous studies have focused on identifying the risk factors for developing a wound complication and ways to mitigate these risks, with varying success.^1,2,3,4,5,6,7,8,11^ For example, a report by Bedi et al^2^ described lower wound complications with vacuum-assisted wound closure in lower extremity sarcomas. A more recent study reported lower wound complication rates with the utilization of indocyanine green fluorescence angiography during wound closure.^11^

In this study, we found notable differences between patients who developed a postoperative wound complication and the patients who did not. For example, markedly more patients in the wound complication cohort had low preoperative albumin, tumors located in the lower extremity, the presence of diabetes, and resections that required flaps for wound coverage during the index surgery. These variables have been shown to be associated with postoperative wound complications, and our findings support these previous studies.^4,8,11^

Compared with the risk factors, the added healthcare costs related to wound complications after a soft-tissue sarcoma surgery are less understood. In other circumstances, wound complications have been shown to increase healthcare costs dramatically, often far exceeding the original treatment.^1,2,13-15^ Surprisingly, this has not been previously analyzed in the soft-tissue sarcoma patient population where wound complications are more frequent and often require more extensive postoperative care. The purpose of our study was to evaluate the increased healthcare costs related to postoperative wound complications in this unique patient population.

When comparing healthcare costs, a notable increase of over 20% was observed when patients developed a postoperative wound complication. This was associated with repeat hospitalizations and additional operations, which most patients required. Each return to the operating room incurred an average incremental cost of 13.2%. Unsurprisingly, the cost was markedly higher when patients developed a wound complication after a free flap or local flap closure during the index procedure as compared to primary closure. This finding, however, is more likely a reflection of the increased complexity and larger soft-tissue defects in these specific cases rather than caused by the type of closure. We continue to use local and free flaps when clinically indicated in our patient population, based on an inability to obtain adequate soft-tissue coverage with primary closure.

In addition to the increased cost, patients who developed a wound complication required more outpatient visits to the orthopaedic surgery and plastic surgery teams during the first postoperative year, as well as almost 6 months of wound care before healing of their wound. This represents a notable time commitment for the patient that is not included in our calculation and is a delay that could affect planned adjuvant treatments.

Our study has several limitations. Namely, it is a retrospective review. The study focuses on a relatively rare disease, and as such, our numbers are limited. Outpatient visits were not included in the cost analysis because many of the visits fell within the 90-day global period after a surgery. Furthermore, several patients received wound care or antibiotic treatment locally, and these charges could not be calculated. In addition, the model does not account for lost revenue because of delays in return to work for the patient. Our model, therefore, underestimates the cost and time commitment required for both the patient and provider when a wound complication is encountered. Despite the limitations, we believe this study helps identify underappreciated challenges in treating patients with sarcoma and demonstrates how reducing wound complication rates may result in notable cost savings for the healthcare system.

## Conclusion

Wound complications after a soft-tissue sarcoma resection are common and add considerable expense to the episode of care. A reduction in wound complications may markedly decrease the cost of treatment and continues to be an opportunity for improvement.
